# Quality of Life in Patients with Acromegaly before and after Transsphenoidal Surgical Resection

**DOI:** 10.1155/2020/5363849

**Published:** 2020-08-04

**Authors:** Jing Gu, Shiyuan Xiang, Min He, Meng Wang, Yanfang Gu, Lingjuan Li, Zhiwen Yin

**Affiliations:** ^1^Department of Nursery, Huashan Hospital, Fudan University, Shanghai 200040, China; ^2^Department of Endocrinology and Metabolism, Huashan Hospital, Fudan University, Shanghai 200040, China

## Abstract

**Objective:**

We aimed to determine the perioperative changes in the quality of life (QoL) in patients with acromegaly and to reveal the relationship between biochemical indicators and quality of life change after tumor resection.

**Methods:**

Patients with acromegaly were enrolled from a tertiary pituitary center. SF-36 scale and AcroQoL scale were used to determine the QoL before and after surgery. We analyzed changes in QoL using a generalized linear model for repeated measurements. We compared the changes in QoL among three groups (remission, active, and discordant group) based on postoperative growth hormone (GH) and insulin-like growth factor-1.

**Results:**

151 patients (75 males and 76 females) diagnosed with acromegaly were included. The average age was 43.9 ± 12.3 years. The median total SF-36 scale was 65.3% (IQR: 63.2%–69.2%). Overall AcroQoL score at baseline was 59.1% (IQR: 51.8%–71.8%). Nadir GH levels (coefficient −0.08, *p*=0.047), T3 levels (coefficient 2.8, *p*=0.001), and testosterone levels (coefficient −0.20, *p*=0.033) in males were independent predictive factors of the total SF-36 score. During the follow-up, the median overall SF-36 score increased to 66.1% at 3 months and 75.3% at 6 months (*p* < 0.001) after surgery. The median overall AcroQoL score increased to 74.5% at 3 months and 77.3% at 6 months (*p* < 0.001) after surgery. At 6-month follow-up, median scores were still less than 70% in appearance, vitality, and mental health dimensions. The QoL after surgery were similar among the three groups, although higher GH and more preoperative somatostatin analogs usage were observed in the active group.

**Conclusion:**

In conclusion, acromegalic patients were associated with low QoL, which could be reversed partially by surgery. The improvement was independent of the endocrine remission. Appearance, vitality, and mental health were three major aspects that warrant further attention from physicians and caregivers after surgery.

## 1. Introduction

Acromegaly, usually caused by a growth hormone-secreting pituitary adenoma, is characterized by increased levels of growth hormone (GH) and insulin-like growth factor-1 (IGF-1). The prevalence of acromegaly in the general population is about 36 to 125 cases per 1 million [1].

The relative risk of death in untreated patients with acromegaly is estimated to be 1.73 compared to the standardized population, which mainly related to cardiovascular, cerebrovascular, and respiratory diseases [2, 3]. Data showed that mortality in patients treated in recent years with modern successful therapies is not different from reference population [4–6]. In addition to that, symptoms of acromegaly include enlargement of face, hands, and feet. Excessive sweat, tiredness, intractable headache, snoring, and sleep apnea are other significant complaints in these patients. Moreover, excessive GH secretion can lead to multiple complications, including diabetes mellitus, hypertension, and osteoporosis. All these together significantly affect the quality of life [7]. Besides, although multimodality treatments can control the disease in most cases, some irreversible changes, such as bone lesions and joint lesions, may still persist [8, 9].

Although there are a large number of cross-sectional studies assessing the quality of life in patients with acromegaly, prospective studies evaluating the changes in the quality of life after surgery-induced remission were limited. In this study, we enrolled 154 patients with acromegaly from January 2018 to July 2019, to examine the perioperative changes in the quality of life using the SF-36 questionnaire and AcroQoL scale. We also analyzed the risk factors associated with the quality of life in these patients before surgery and explored the relationship between biochemical changes and quality of life change after tumor resection.

## 2. Methods

Inpatients diagnosed with acromegaly were enrolled from the neurosurgical department of Huashan Hospital, Fudan University from January 2018 to July 2019. The diagnosis of acromegaly was based on typical clinical features of acromegaly, high GH after oral administration of 75 g glucose (OGTT), high IGF-1 levels [7, 10], pituitary tumor confirmed by MRI, and the pathology. We only included newly diagnosed acromegalic patients who were submitted to transnasal transsphenoidal surgery. We excluded adult patients without sufficient follow-up. The institutional review board approved the study, and all patients gave informed consent.

We documented baseline characteristics of the cohort, including age, gender, body mass index, medical history, and education history. Endocrinological tests were performed before operation. GH levels were analyzed using a two-site chemiluminescence immunoassay (AutoDELFIA® hGH, PerkinElmer Life and Analytical Sciences, Wallac Oy) with intra-assay variance of 5.3–6.5%, interassay variance of 5.7–6.2%, and sensitivity of 0.01 *μ*g/l. IGF-1 levels were analyzed using Immulite 2000 solid phase, enzyme-labeled chemiluminescence immunoassay analysis (Siemens Medical Diagnostics Ltd., United Kingdom). Normal age-appropriate ranges of IGF-1 for adults are as follows: 19-20 years: 127–483 *μ*g/l; 21–35 years: 115–358 *μ*g/l; 36–50 years: 94–284 *μ*g/l; >50 years: 55–238 *μ*g/l. The intra-assay variance of IGF-1 was 2.3–3.5%, the interassay variance was 7.0-7.1%, and the sensitivity of IGF-1 was 20 *μ*g/l [11]. Hormones of the thyroid axis, the adrenal axis, and the gonadal axis were also tested before surgery.

### 2.1. Questionnaires

We used SF-36 and AcroQoL questionnaires to assess the quality of life in patients with acromegaly.

The SF-36 scale is currently the most widely used life quality assessment tool in the world. The scale includes nine dimensions, namely, physical functioning, role physical, bodily pain, general health, vitality, role emotional, social functioning, mental health, and health transition. We applied the standardized scale method to scale each dimension and the total SF-36 score. We presented the scores as percentages, and higher score represents better quality of life [12].

The AcroQoL is the only evaluation scale specifically for the health-related quality of life in patients with acromegaly. The scale includes 22 items, mainly evaluating three dimensions: patients' physical function, appearance, and personal relationship [13]. The scale is suitable for patients with acromegaly between 18 and 70 years old, using a 5-grade scoring method. Similarly, scores were presented as percentages in each dimension and the total AcroQoL score.

### 2.2. Statistical Analysis

Continuous data were described as mean ± standard deviation (interquartile range for nonnormalized data), and the count data were described as count and proportion. Correlations among baseline characteristics and scores in quality of life were assessed using Spearman correlation tests. Linear regression was used to identify independent risks for the poor quality of life.

Change in quality of life was analyzed using a generalized linear model for repeated measurements. We further analyzed the change in quality of life among three groups according to the 2010 consensus: remission group (random GH < 1ug/L or nadir GH < 0.4 *μ*g/L during an OGTT, and the IGF-1 index < 1), active group (random GH > 1 *μ*g/L or nadir GH > 0.4 *μ*g/L during an OGTT, and IGF-1 index > 1), and discordant group (GH criteria and IGF-1 criteria were discordant). We used *R* v3.3.2 to perform statistical analysis and *p* < 0.05 as the significant level.

## 3. Results of Baseline Characteristics

151 patients (75 males and 76 females) diagnosed with acromegaly were included, and the average age was 43.9 ± 12.3 years. The most common comorbidity was hypertension (40 patients, 26.5%) and diabetes mellitus (38 patients, 25.2%). 29 patients (19.2%) received somatostatin analogs treatment before surgery. Regarding education history, 97 patients only experienced undergraduate education, 51 patients accepted college or university education, and 3 patients underwent a postgraduate education.

The median total SF-36 scale was 65.3% (IQR: 63.2%–69.2%, Table 1). Role emotional, role physical, and general health were the poorest three dimensions with 33.3% (IQR: 0.0%–100.0%), 50.0% (IQR: 25.0%–75.0%), and 45.0% (IQR: 27.0%–57.0%), respectively. Bodily pain, vitality, and mental health were compromised as well, with 74.0% (IQR: 74.0%–80.0%), 70.0% (IQR: 50.0%–75.0%), and 72.0% (IQR: 52.0%–72.0%), respectively. Physical functioning and social functioning subscales were relatively normal. Overall AcroQoL score at baseline was 59.1% (IQR: 51.8%–71.8%). The median physical subscore was 65.0% (IQR: 47.5%–77.5%), the median appearance subscore was 57.1% (IQR: 48.6%–62.9%), and the median personal relation subscore was 74.3% (IQR: 62.9%–80.0%).

**Figure 1:**
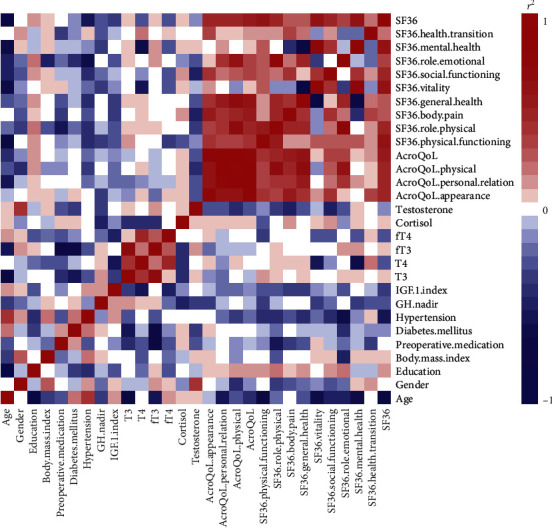
Correlation plot among baseline characteristics and quality of life scores. Red represents positive correlation and blue represents negative correlation.

We observed that education history slightly positively correlated with quality of life (Figure 1). Regarding negative factors, age and hypertension negatively correlated with both SF-36 and AcroQoL. Additionally, within the dimensions in AcroQoL, all subscales of AcroQoL correlated with each other. Within the dimensions of SF-36, mental health and vitality slightly negatively correlated with general health. Figure 1 summarizes the correlations among various acromegaly-related parameters and the quality of life scores.

In the multiple linear regression analysis model, we found that nadir GH levels (coefficient −0.08, *p*=0.047), T3 levels (coefficient 2.8, *p*=0.001), and testosterone levels (coefficient −0.20, *p*=0.033) in males were independent predictive factors of total SF-36 score. No factors were observed to be correlated with total AcroQoL score, but age was associated with personal subscale (coefficient −0.11, *p*=0.008) and appearance subscale in AcroQoL (coefficient −0.06, *p*=0.038).

### 3.1. Postoperative Changes in Quality of Life

During the follow-up, the median overall SF-36 score increased to 66.1% at 3 months and 75.3% at 6 months (*p* ≤ 0.001) after surgery. The median overall AcroQoL score increased to 74.5% at 3 months and 77.3% at 6 months (*p* ≤ 0.001) after surgery. We found that all AcroQoL subscales and SF-36 subscales increased, except for the SF-36 vitality, social functioning, and mental health subscales, in which no differences were found at follow-up compared with baseline. At 6-month follow-up, median scores were still less than 70% in appearance, vitality, and mental health dimensions.

We found that the baseline quality of life was associated with the life quality change after surgical resection (coefficient −1.28, *p*=0.020 for SF-36 and coefficient −1.20, *p*=0.003 for AcroQoL), suggesting that patients with low quality of life at baseline were more likely to recover after surgery.

Further analyses were performed among the remission group, active group, and discordant group (Table 2). The quality of life after surgery was similar among the three groups, although higher GH and more preoperative somatostatin analogs usage were observed in the active group.

## 4. Discussion

We prospectively enrolled patients with acromegaly and investigated their quality of life in this study. We found that the quality of life was compromised in patients with acromegaly, with growth hormone, triiodothyronine, and testosterone in males as risk factors for low quality of life. Quality of life increased after surgery, but subscores of appearance, vitality, and mental health dimensions were still low after surgery. The change was not dependent on the endocrine remission of the disease.

Quality of life assessment is an important patient-reported outcome measurement in clinical practice. It reflects the overall disease course and remains pertinent to many other aspects such as mental state and social activities. The relationship between growth hormone excess and quality of life is complicated, due to the limitations of our current understanding of acromegaly [14]. The understanding of quality of life improving in patients after transnasal pituitary tumor resection can potentially improve physicians' clinical care and patients' well-being.

Biermasz et al. found that the disease course and age negatively correlated with the AcroQoL score, which was similar to our findings [15]. Dimopoulou et al. found that headache, GH levels, depression, and the decline in quality of life were related. Treatment for depressive symptoms may improve the patients' overall quality of life [16]. Miller et al. found that patients with musculoskeletal pain had poorer quality of life compared with those without pain in a cross-sectional study of patients with chronic acromegaly [17]. In regard of endocrine functions, Geraedts et al. found endocrine functions are not associated with quality of life [14]. However, in our study, we found that pituitary insufficiency was an independent risk factor for poor quality of life.

Concerning the subscales, Kyriakakis et al. found that physical function was the most significant subscale in SF-36, which might be related to irreversible complications of the joint disease [18]. Webb et al. found that the appearance dimension weakly correlated with the GH level. Appearance and physiological function were the most affected areas in AcroQoL [19], which corresponds to our findings.

Patients had a significant improvement in the quality of life after surgery, according to several studies. Badia et al. found that the quality of life in patients who achieved biochemical control was better than those who did not achieve biochemical control [20, 21]. However, in another study conducted by Webb and Badia [19], biochemical control does not necessarily translate into the patient's perception of normal quality of life [22, 23]. Our study concluded similarly that although biochemical parameters were not always normal, successful surgery could improve the overall health of the patient even if he/she remained active after surgery. Similarly, Webb and Bandia found no difference in the quality of life scores between active and inactive patients [19].

In this study, we did not find sufficient evidence to support the benefit of biochemical control on improving patients' quality of life, which suggested that biochemical control was not the only choice in quality of life measurement. The dimensions that had most significant impact on the patient's quality of life at 6-month follow-up were appearance, mental health, and vitality. Further microplastic surgeries, treatment of depressive symptoms, and sufficient endocrine replacement have a promising strategy for improving quality of life [14]. Physicians and caregivers should pay more attention to the long-term mental and psychological problems of acromegalic patients and to ensure their effective management of comorbidities.

The study's major limitation lies in that we only evaluated patients in a relatively short period; the long-term quality of life in acromegalic patients was unknown. Different treatment modes have different effects on the quality of life, and we were unaware of the quality of life after medical or radiosurgical therapy. Occupational information and financial information were not available. Questionnaire response behavior can also be biased by current mood or context effects; for example, patients' quality of life may be compromised by the complications of surgery (e.g., nasal congestion) during the short term after surgery. Though similar results have been published in previous studies, our proof-of-concept result suggested that further microplastic surgeries, treatment of depressive symptoms, and sufficient endocrine replacement might have a promising strategy for improving quality of life in these patients. Future studies should focus on intervention.

In conclusion, low quality of life in acromegalic patients could be reversed partially by surgery. The improvement was independent of the endocrine remission. Appearance, vitality, and mental health were three major aspects that warrant further attention from physicians and caregivers after surgery.

## Figures and Tables

**Table 1:** Quality of life before and after operation in acromegalic patients.

	Baseline (%)	3-month follow-up (%)	6-month follow-up (%)	*p* for repeated measure
SF-36	65.3 (63.2–69.2)	66.1 (64.9–72.8)	75.3 (63.9–83.2)	≤0.001
Physical functioning	90.0 (90.0–95.0)	85.0 (85.0–95.0)	90.0 (80.0–100.0)	0.001
Role physical	50.0 (25.0–75.0)	25.0 (25.0–75.0)	75.0 (75.0–100.0)	≤0.001
Bodily pain	74.0 (74.0–80.0)	84.0 (74.0–100.0)	72.0 (70.0–84.0)	≤0.001
General health	45.0 (27.0–57.0)	75.0 (52.0–82.0)	67.0 (50.0–77.0)	≤0.001
Vitality	70.0 (50.0–75.0)	45.0 (40.0–70.0)	65.0 (55.0–75.0)	0.669
Role emotional	33.3 (0.0–100.0)	66.7 (66.7–100.0)	100.0 (66.7–100.0)	≤0.001
Social functioning	100.0 (100.0–100.0)	87.5 (62.5–100.0)	100.0 (100.0–100.0)	0.894
Mental health	72.0 (52.0–72.0)	56.0 (56.0–68.0)	68.0 (56.0–80.0)	0.170
Health transition	50.0 (25.0–50.0)	50.0 (50.0–75.0)	75.0 (50.0–75.0)	≤0.001
AcroQoL	59.1 (51.8–71.8)	74.5 (70.0–76.4)	77.3 (59.1–87.5)	≤0.001
Appearance	57.1 (48.6–62.9)	54.3 (54.3–60.0)	62.9 (54.3–82.9)	≤0.001
Personal relation	74.3 (62.9–80.0)	91.4 (77.1–91.4)	82.9 (65.7–91.4)	≤0.001
Physical	65.0 (47.5–77.5)	77.5 (72.5–82.5)	82.5 (62.5–90.0)	≤0.001

**Table 2:** Difference of quality of life change in remission, discordant, and active patients.

	Remission	Discordant	Active	*p*
	(*N* = 71)	(*N* = 36)	(*N* = 44)	
Baseline characteristics				
Age (years)	45.3 (12.5)	41.5 (11.2)	43.6 (12.9)	0.318
Gender (male)	34 (47.9%)	17 (47.2%)	24 (54.5%)	0.743
BMI (kg/m^2^)	25.4 (2.9)	25.5 (3.3)	26.7 (4.2)	0.122
Preoperative medication	8 (11.3%)	7 (19.4%)	14 (31.8%)	0.025
Diabetes mellitus	17 (23.9%)	11 (30.6%)	10 (22.7%)	0.687
Hypertension	22 (31.0%)	4 (11.1%)	14 (31.8%)	0.056
Nadir GH (ng/mL)	15.7 (13.6)	20.3 (14.9)	27.8 (24.6)	0.004
IGF index	2.5 (1.0)	2.3 (0.8)	2.7 (0.9)	0.178
T3 (nmol/L)	2.1 (1.3)	2.1 (1.7)	2.2 (1.7)	0.949
T4 (nmol/L)	94.8 (23.4)	94.0 (24.2)	97.0 (25.1)	0.857
Free T3 (pmol/L)	5.3 (1.6)	5.2 (1.9)	8.0 (17.5)	0.307
Free T4 (pmol/L)	18.4 (12.6)	18.7 (17.5)	18.1 (8.8)	0.979
Testosterone (nmol/L)	7.8 (3.8)	14.5 (25.3)	6.1 (7.2)	0.111
SF36	65.6% [63.9%, 72.1%]	65.3% [62.1%, 66.6%]	65.4% [63.2%, 67.7%]	0.521
AcroQoL	58.2% [51.8%, 71.8%]	67.7% [51.8%, 71.8%]	64.1% [51.8%, 71.8%]	0.982
6-month follow-up				
SF36	76.1% [61.4%, 81.9%]	78.2% [63.7%, 88.9%]	75.3% [70.1%, 82.3%]	0.431
AcroQoL	75.0% [59.3%, 87.3%]	76.4% [57.3%, 89.3%]	82.7% [74.1%, 88.6%]	0.335
Change of QoL after surgery				
SF36	11.1% [−1.5%, 16.1%]	18.7% [−0.7%, 35.7%]	11.0% [2.5%, 20.7%]	0.384
AcroQoL	7.0% [2.2%, 31.0%]	14.5% [5.8%, 24.4%]	15.4% [2.4%, 39.6%]	0.647

## Data Availability

The data used to support the findings of this study are available from the corresponding author upon request.

## References

[1] Capatina C., Wass J. A. H. (2015). 60 years of neuroendocrinology: acromegaly. *Journal of Endocrinology*.

[2] Dekkers O. M., Biermasz N. R., Pereira A. M., Romijn J. A., Vandenbroucke J. P. (2008). Mortality in acromegaly: a meta analysis. *The Journal of Clinical Endocrinology & Metabolism*.

[3] Orme S. M., McNally R. J. Q., Cartwright R. A., Belchetz P. E. (1998). Mortality and cancer incidence in acromegaly: a retrospective cohort study1. *The Journal of Clinical Endocrinology & Metabolism*.

[4] Esposito D., Ragnarsson O., Granfeldt D., Marlow T., Johannsson G., Olsson D. S. (2018). Decreasing mortality and changes in treatment patterns in patients with acromegaly from a nationwide study. *European Journal of Endocrinology*.

[5] Maione L., Brue T., Beckers A. (2017). Changes in the management and comorbidities of acromegaly over three decades: the French acromegaly registry. *European Journal of Endocrinology*.

[6] Bolfi F., Neves A. F., Boguszewski C. L., Nunes-Nogueira V. S. (2018). Mortality in acromegaly decreased in the last decade: a systematic review and meta-analysis. *European Journal of Endocrinology*.

[7] Katznelson L., Laws E. R., Melmed S. (2014). Acromegaly: an endocrine society clinical practice guideline. *The Journal of Clinical Endocrinology & Metabolism*.

[8] Biermasz N. R., van Thiel S. W., Pereira A. M. (2004). Decreased quality of life in patients with acromegaly despite long-term cure of growth hormone excess. *The Journal of Clinical Endocrinology & Metabolism*.

[9] Claessen K. M. J. A., Mazziotti G., Biermasz N. R., Giustina A. (2016). Bone and joint disorders in acromegaly. *Neuroendocrinology*.

[10] Katznelson L., Atkinson J., Cook D. (2011). American association of clinical endocrinologists medical guidelines for clinical practice for the diagnosis and treatment of acromegaly-2011 update. *Endocrine Practice*.

[11] Shen M., Wang M., He W. (2018). Impact of long-acting somatostatin analogues on glucose metabolism in acromegaly: a hospital-based study. *International Journal of Endocrinology*.

[12] Ware J. E., Gandek B. (1998). Overview of the SF-36 health survey and the international quality of life assessment (IQOLA) project. *Journal of Clinical Epidemiology*.

[13] Webb S. M., Prieto L., Badia X. (2002). Acromegaly quality of life questionnaire (ACROQOL) a new health-related quality of life questionnaire for patients with acromegaly: development and psychometric properties. *Clinical Endocrinology*.

[14] Geraedts V. J., Andela C. D., Stalla G. K (2017). Predictors of quality of life in acromegaly: no consensus on biochemical parameters. *Front Endocrinology (Lausanne)*.

[15] Biermasz N. R., Pereira A. M., Smit J. W. A., Romijn J. A., Roelfsema F. (2005). Morbidity after long-term remission for acromegaly: persisting joint-related complaints cause reduced quality of life. *The Journal of Clinical Endocrinology & Metabolism*.

[16] Dimopoulou C., Athanasoulia A. P., Hanisch E. (2014). Clinical characteristics of pain in patients with pituitary adenomas. *European Journal of Endocrinology*.

[17] Miller A., Doll H., David J., Wass J. (2008). Impact of musculoskeletal disease on quality of life in long-standing acromegaly. *European Journal of Endocrinology*.

[18] Kyriakakis N., Lynch J., Gilbey S. G., Webb S. M., Murray R. D. (2017). Impaired quality of life in patients with treated acromegaly despite long-term biochemically stable disease: results from a 5-years prospective study. *Clinical Endocrinology*.

[19] Webb S. M., Badia X. (2016). Quality of life in acromegaly. *Neuroendocrinology*.

[20] Paisley A. N., Rowles S. V., Roberts M. E. (2007). Treatment of acromegaly improves quality of life, measured by AcroQol. *Clinical Endocrinology*.

[21] Trepp R., Everts R., Stettler C. (2005). Assessment of quality of life in patients with uncontrolled vs. controlled acromegaly using the acromegaly quality of life questionnaire (AcroQoL). *Clinical Endocrinology*.

[22] Hua S.-C., Yan Y.-H., Chang T.-C. (2006). Associations of remission status and lanreotide treatment with quality of life in patients with treated acromegaly. *European Journal of Endocrinology*.

[23] T’Sjoen G., Bex M., Maiter D., Velkeniers B., Abs R. (2007). Health-related quality of life in acromegalic subjects: data from AcroBel, the Belgian registry on acromegaly. *European Journal of Endocrinology*.

